# 

**Published:** 1888-06-02

**Authors:** 


### AN APPEAL ON THE FACTS.

It will be observed that 1,259,512 separate persons received treatment and relief at the hospitals and dispensaries of London during the year 1887. The population of London, as estimated by the Registrar-General on October 1st, 1887, was 4,215,192, so that at least one in every four persons, and probably one in every three of the whole population, visited the hospitals during some period of the year 1887 for the purpose of obtaining medical advice or medicine. The total money value, as represented by the actual expenditure on the relief thus afforded, was £651,802, or something like 10s. 5d. per person relieved.

Towards defraying the cost to the hospitals of directly relieving in a single year upwards of one million and a-quarter of sick people, the charitable and proprietary revenue, with the patients' payments contributed, were inadequate to the extent of £100,000, a deficiency which would have been half as much again, except for the money at present subscribed on Hospital Sunday. We ask the wealthy, the thoughtful, and the generous to study the above figures. They will show to all how each charity does its work, and what is the principle upon which that work is carried on.

Some people hold, with Mr. Spurgeon, that the managers of a charity should never be waiters upon Providence, but that funds should be collected and placed in the bank before orders are given for the work to be undertaken. This list will be found to contain institutions which have managed to make both ends meet and a little over, so that Mr. Spurgeon's principles are well represented. Unfortunately, however, it is not always possible to conduct a hospital on such business-like principles, or many sick and suffering folk would fare badly indeed at times. To be of the greatest utility, a hospital has often to be placed in the midst of a dense and indigent population, who have not the means of supporting it adequately, and the managers have therefore to appeal to the charitable who live in the wealthier parts of the town. In such circumstances it necessarily happens that the hospital is in a chronic state of impecuniosity, and many such institutions will be found with the others given here. It is not too much to say that the very varying condition of the hospitals of London revealed in these Tables must include every conceivable variety of circumstance, and so be calculated to meet the views of every group of philanthropists. This fact should call forth an amount of enthusiasm worthy of so great a cause, and the pressing pecuniary needs of a class of charities, the efficient maintenance of which is a matter of vital importance to all classes of the community. At least £100,000 is needed. Shall it not be forthcoming? Will the flowers of so fragrant a garden be neglected by men and women who have means? The receipts on Hospital Sunday will afford the best answer. In London to-day

> "The charities which soothe, and heal, and bless,
> Lie scattered at the feet of men like flowers."

Who will not gather a button-hole at least, if a bouquet be indeed beyond their resources?

